# Depressive, Anxious, and Post-Traumatic Stress Symptoms Related to Violence during the COVID-19 Pandemic, by Sex, COVID-19 Status, and Intervention-Seeking Conditions among the General Population

**DOI:** 10.3390/ijerph191912559

**Published:** 2022-10-01

**Authors:** Silvia Morales Chainé, Rebeca Robles García, Alejandro Bosch, Claudia Lydia Treviño Santa Cruz

**Affiliations:** 1 Facultad de Psicología, Universidad Nacional Autónoma de México, Mexico City 04510, Mexico; 2 Instituto Nacional de Psiquiatría “Ramón de la Fuente Muñiz”, Mexico City 14370, Mexico; 3 Dirección General de Atención a la Comunidad, Universidad Nacional Autónoma de México, Mexico City 04510, Mexico; 4 Instituto de Biotecnología, Universidad Nacional Autónoma de México, Cuernavaca 62210, Mexico

**Keywords:** depression, anxiety, posttraumatic stress, violence, COVID-19, help-seeking, measurement

## Abstract

Worldwide, three out of four of the general population have reported experiencing violence. Governments should address solutions to violence and its effects on mental health. The study aimed to determine depressive, anxious, and posttraumatic stress symptoms related to the violence experienced during the COVID-19 pandemic in the general population. The study was conducted with 18,449 Mexicans of 33 years (SD = 11.00, range = 18–59), with 12,188 (66.10%) being women, 3559 (19.29%) having COVID-19, 2706 (14.67%) seeking psychological care, and 5712 (30.96%) experiencing violence. Subjects completed the Major Depressive Episode (MDE) Checklist, Generalized Anxiety (GA) Scale, and the Posttraumatic Stress (PTS) Checklists (PCL-5) programmed in a WebApp application. We assessed the dimensionality of the scales through the Confirmatory Factor Analysis (CFA), the measurement invariance, and a structural equation model (SEM). In the total sample, 28.10% fulfilled the MDE criteria, and 42.30% had high levels of GA. In the sample of those experiencing violence, 48.40% met the MDE criteria, 61.70% had high GA symptoms, and 50% met the criteria for a PTS disorder. Experiencing violence was associated with GA and severe PTS symptoms when the discomfort had bothered them for over a month since the onset of these symptoms. Subjects who had experienced violence and had mental health symptoms seemed ready for treatment. Further studies will evaluate the effect of remote psychological care to help reduce the treatment gap.

## 1. Introduction

Since the onset of the COVID-19 pandemic, the risk of suffering from the infection has been associated with the prevalence of depression, anxiety, and stress symptoms. Bourmistrova et al. [1] recently reported the prevalence of mental health symptoms at follow-up assessment associated with contracting COVID-19. Their systematic review indicated a prevalence of 18.85% of depression and 20.84% three months after having COVID-19. These authors also reported a 19.03% prevalence of anxiety one month after being infected with COVID-19 and a 11.11% prevalence at a three-month follow-up. They also reported a 17.68% prevalence of posttraumatic stress disorder (PTSD), and a 12.19% and 18.99% prevalence, at one- and three-month follow-ups, respectively. 

Earlier in 2020, Rogers et al. [2] reported that survivors of SARS MERS and Ebola in previous epidemics suffered from at least one mental health symptom. However, the study by Bourmistrova et al. [1] indicated that long-term mental health symptoms should be attributed more to psychosocial factors during COVID-19 than the infection itself. The assessment of conditions related to the presence of mental health conditions during the COVID-19 pandemic was therefore described as essential.

The World Health Organization (WHO [3]) emphasized the importance of psychosocial factors, when it indicated that violence posed a major threat to global public health during the COVID-19 pandemic. It was reported that one in three women worldwide experienced physical and/or sexual violence by an intimate partner. In Mexico, we assessed 9361 adults, 71% of whom were women, and suggested that acute stress, avoidance, sadness, distancing, anger, and generalized anxiety symptoms were related to experiences of emotional and physical abuse [4].

Parallel findings have indicated that women reported depressive, anxious, and hyperarousal (stress) symptoms during the COVID-19 pandemic. A path of symptoms was characterized by depression predicting hyperarousal symptoms, avoidance predicting reexperiencing, and depression and avoidance predicting negative cognitive and emotional stress symptoms (NACM) [5]. These results suggest evidence of a time lapse between the occurrence of traumatic events and the development of stress symptoms, in addition to those involving depression and anxiety.

In their description of the psychometric evaluation of the Posttraumatic Check List (PCL-5) (DSM-5; American Psychiatric Association (APA, 201300) [6,7] reported that depression and anxiety are associated with PTSD symptoms in college students who have suffered traumatic experiences including violence. 

To assess trauma as a stressful experience, Blevins et al. [7] include blame, negative emotions, and reckless/self-destructive behavior in the recent PCL-5 and validated its factor structure. They suggested that PTSD was a latent variable separate from anxiety and depression, yet strongly associated with them. 

In another study, we validated depressive-anxiety-stress symptom scales during the COVID-19 pandemic [5]. We reported four latent stress variables: intrusion/reexperiencing, avoidance, hyperarousal, and numbing symptoms from the PCL-C (based on the DSM, 4th edition [8,9], in addition to depression and anxiety [10] in the general population. Those scales were validated through Confirmatory Factor Analysis (CFA) and the goodness of fit of the model (Comparative Fit Index (CFI), Tucker–Lewis Index (TLI), Root Mean Square Error of Approximation (RMSEA), and Standardized Root Mean Square Residual [SRMR] [11,12]. We reported the invariance measurement [13] of Goldberg’s anxiety scale and the PCL-C to identify bias while comparing symptoms by groups of interest (such as sex). One of the important comparisons was therefore between subjects seeking psychological care versus those who were not. 

In 2015, Blevins and collaborators reported good PCL-5 fit indices in the four factor-structure model, in keeping with the DSM-5 [6]. Authors provided evidence of the internal consistency of the PCL-5, its convergent-discriminant validity, and structural validity based on CFA with 836 subjects, 73.05% females and 19.7% reporting physical-sexual assault. However, Blevins et al. [7] strongly recommended assessing the response bias and semantic overlap between items while working with the general population and comparing men and women in relation to a specific trauma.

Consequently, the context in which mental health tools are administered, and their assessment of measurement invariance, as suggested by Elhai & Palmieri [14], are actions that should reveal the biases between the groups compared [13] (such as sex]. These actions should guide decision-making regarding the screening of mental health symptoms that could vary because of the characteristics of the population (community vs. specialized settings), or the type of traumatic events to which they have been exposed, such as abuse [15]. 

As a tool for assessing measurement invariance (metric, strong, rigorous) by comparing samples (such as the experience of abuse), CFA generates evidence of the structural stability of the assessment. It is therefore possible to compare groups by sociodemographic or cultural factors, specific traumatic events, or willingness to accept intervention [5]. The structure factor of mental health screening and its fitted model analyses are justified when researchers work with different traumas, such as abuse experienced during the COVID-19 pandemic e.g., [7]. Valid findings should be considered when physical, emotional, or sexual abuse experiences are reported and when people are aware that they require psychological care to reduce the treatment gap [Mental Health Gap Action Programme (mhGAP) [16]. 

It is therefore essential to assess the association between various psychosocial factors and the principal mental health symptoms [1] by analyzing the measurement invariance of the instrument and the scope of the comparisons through a verified structure factor of the Major Depressive Episode (MDE) (DSM-5; [6]), the Generalized Anxiety (GA) Scale [10], and the PCL-5. Accordingly, the purpose of this study was to determine depressive, anxious, and posttraumatic stress (PTS) symptoms related to the experience of abuse during the COVID-19 pandemic in the general population by validating an electronic tool and obtaining its measurement invariance by sex, COVID-19 status, psychological care-seeking, the experience of violence, and persistent stressful symptoms. A structural equation model (SEM) was used to analyze the path of latent mental-health variables with the sample that had experienced violence e.g., [5].

## 2. Method

### 2.1. Design 

In this correlational study, subjects were invited to enroll in a programmed platform, WebApp 2.6.1 version (programmed in Linux^®^, PHP^®^, HTML^®^, CSS^®^, and JavaScript^®^-React^®^-Node^®^ softwares by DGACO-UNAM, Mexico City, Mexico [17], see Appendix A), between 13 December 2020 and 31 August 2021. The link was available through the Mexican Health Ministry Website (announced by press conferences on the radio, television, and Internet).

### 2.2. Subjects

We surveyed 18,449 subjects with a mean age of 33, (SD = 11.00, range = 18–59), 12,188 (66.10%) of whom were women, 3559 (19.29%) of whom had tested positive for COVID-19, 2706 (14.67%) of whom were seeking psychological care, and 5712 (30.96%) of whom had reported experiencing violence. The distribution of the total sample by comparison variables is given in Table 1. This table also shows the distribution of subjects’ experience of violence per variable.

Subjects agreed to answer the survey in keeping with the privacy policies established in the General Protection of Personal Information in Possession of Obligated Parties Act [Spanish Acronym LGPDPPSO] [18], and the General Office of the Community Care Guidelines of the National Autonomous University of Mexico (Spanish Acronym DGACO-UNAM). Data were asymmetrically encrypted. The database was held in the official university domain, with security locks to protect the information and guarantee its management in keeping with the subjects’ informed consent.

In the informed consent form, researchers told subjects that confidentiality would be maintained by calculating general averages. Subjects were told that they would be used for dissemination and epidemiological research and that they had the right to decline the use of their information and drop out at any point in the study. Although incentives were not offered, immediate feedback was supplied in the form of psychoeducational tools (infographics, videos, and Moodle^®^ courses on COVID-19, self-care, relaxation techniques, problem-solving, and socioemotional management skills). Phone numbers were provided to obtain remote psychological care from the Health Ministry and UNAM Services. Finally, the benefits of accessing the platforms or requesting help with dealing with mental health conditions were described. A data section, in which subjects were able to give their phone numbers or email so that they could be contacted, was included to enable them to request remote psychological care. The protocol was approved with the code FPSI/422/CEIP/157/2020 by the Institutional Review Board of the UNAM Psychology Faculty Ethics Committee on Applied Research on 16 October 2020.

### 2.3. Instruments 

For this study, a WebApp comprising four instruments, described in Appendix A, was operated with the systems and program languages Linux^®^, PHP^®^, HTML^®^, CSS^®^, and JavaScript^®^-React^®^-Node^®^ [17] (See https://www.misalud.unam.mx/covid19/ (accessed on 15 August 2022)) to the proper understanding of the WebApp). First, three dichotomic responses (yes or no) questions about sex, COVID-19 status, and remote psychological care-seeking were included. 

Second, the WebApp included the 11 five-option-response items from the MDE checklist (DSM-5 [6], see Appendix A). The response options involved indicating how often subjects had experienced the symptoms in in the past twelve months: always (1), nearly always (2), sometimes (3), rarely (4), or never (5). To calculate the total score, we considered several steps: part 1, part 2, part 3, criterion A and B guidelines. The criteria for Part 1 were met when items one and two (*Sadness or depressed mood?* and *Discouraged because of how things are going in your life?*) were answered with options 1 or 2. The criteria for Part 2 were met when five or more items were answered with options 1 or 2 from items 2 to 10 plus part 1. The criteria for Part 3 were met when question 3 (*Loss of interest or pleasure?*) was recorded with response options 1 or 2. Criterion A was met when part 1 and part 2 or 3 were completed. Criterion B was met when question 11 (*Symptoms causing impairment in social, occupational, or other important areas of functioning?*) was recorded with response options 1, 2, or 3. Finally, an MDE was identified when criteria A and B were met [6]. 

Third, the WebApp included the GA scale (adapted from [5] and Goldberg et al. [10]; see Appendix A), comprising five items with eleven response options. The response options ranged from zero (total absence of the symptom) to ten (total presence of the symptom) in the question about whether subjects had felt anxious in the past two weeks. We therefore screened for GA by adding the score and dividing it by five. In keeping with the Goldberg et al. [10] study, an average of 60% was considered a high level of GA. 

Appendix B shows the SEM based on the MDE and GA items, their factor loadings, covariances from modification indices (MI), latent variables, residual variance, standard errors, regression coefficients, and overall model fit indices ($X^{2}$(86) = 4713.12, *p* = 0.000, a *CFI* = 0.977, a *TLI* = 0.972, an *RMSEA* = 0.054 [confidence intervals of 0.053–0.055], and an *SRMR* = 0.025) for the total sample in this study. The Cronbach’s alphas of the scales were 0.92 and 0.94 for MDE and GA, respectively. 

Fourth, the WebApp included the 11 yes/no-dichotomic-response items from the checklist on the experience of violence in past six months, adapted from the PCL-5 criterion A [19] (Life Events Checklist for DMS-5-LEC-5, see Appendix A). If subjects checked any of the experience of violent events in Criterion A, they were asked to choose the one that bothered them most at the time. If they had only had one experience of violence, they were asked to choose the most severe event to answer the questions in part B of PCL-5. 

The 20 five-option-response items in the PCL-5 (criterion B, C, and D; see Appendix A) were included in the electronic tool [7,20]. Responses were: not at all (0), slightly (1), moderately (2), quite a lot (3), and extremely (4) bothersome symptoms in the past month. We used the four-factor DMS-5 [6] structure [7]: reexperiencing with five items, avoidance with two items, NACM with seven items, and hyperarousal with six items. In the WebApp, we included the less/more-than-a-month response for how long have the symptoms been bothering the subject.

Blevins et al. [7] reported that the four-factor structure was a model with a good fit ($X^{2}$ (164) = 558.18, *p* < 0.001, a *CFI* = 0.91, a *TLI* = 0.89, an *RMSEA* = 0.07, and an *SRMR* = 0.05; alpha = 0.94), whose optimal score of 31 (from the total of 80) yielded a sensitivity of 0.77, a specificity of 0.96, an efficiency of 0.93 and a quality of efficiency of 0.73.

In addition, the PTSD criterion was considered when a subject selected a 2-response option or more for at least one of the B-items, one of the C-items, two of the D-items or two of the E-items, and symptoms had been bothering them for over a month. 

### 2.4. Data Analysis

The statistical procedure involved several analytical steps. First, we examined the dimensionality of the MDE and GA scales to provide their construct validity evidence for the total sample. We used the 2-factor CFA model from maximum likelihood to continuous variable data as an estimation method [14], and the SEM of the latent variables (see Appendix B). The overall fit of the model was assessed using the chi-square goodness of fit test. Since the chi-square goodness of fit test is over-sensitive to large sample sizes, more emphasis was given to fit indices such as the *CFI*, *TLI*, *RMSEA*, and *SRMR*. Models with *CFI* and *TLI* values greater than 0.90 and *RMSEA* and *SRMR* values of less than 0.08 and 0.06, respectively, were considered indicators of adequate data fit [11,12]. We obtained the MDE and GA reliability with the Cronbach’s Alpha test and the correlations between them with the Spearman test to determine the degree of association and independence between the dimensions.

Second, we analyzed the measurement invariance for MDE and GA by comparing sex, COVID-19 status, psychological care-seeking, and the experience of violence in the groups to ascertain the extent to which the items showed equivalent psychometric properties for the total sample. A series of multiple-group CFA models fit the data, each with an increasing number of equality constraints in the item parameters [21,22,23]. 

Measurement invariance involved calculating configural, metric, strong, and strict invariances. Configural invariance was tested by allowing all parameters (loadings, thresholds, and unique factor variances) to be freely estimated. Next, metric invariance was assessed by constraining the item loadings to equality across comparison groups. Strong measurement invariance was tested by constraining the item thresholds to equality across comparison groups. Finally, strict measurement invariance tested equality across comparison groups in the unique variances, including the unique variances of the correlations between the pairs of items referred to by the MI in CFA. Nested models were evaluated using the chi-square test for continuous data. We also examined the *CFI* and *TLI* change from the less restricted model to the more constrained one (Δ). The more constrained model with changes in the *CFI* values of 0.010 or less was considered acceptable [24], and *RMSEA* values of 0.015 or less were also considered acceptable. In cases where the invariance models did not fit the data, partial invariance was examined by allowing some of the item parameters to vary between groups. The LavTestScore and ParTable commands (LTS-PT) were examined to determine which item parameters needed to be freely estimated across groups. The measurement invariances were calculated for each comparison group of the study (such as sex).

Third, we examined the dimensionality of the MDE, GA, and the four-factor PTSD scales (reexperiencing, avoidance, NACM, and hyperarousal) to provide their construct validity evidence for the abuse experience sample through the CFA and the SEM of the latent variables. In this way, we obtained the MDE, GA, and PTSD factor reliability with the Cronbach Alpha test and the correlations between them with the Spearman test to determine the extent of the association and the independence between the dimensions for this sample.

Fourth, we analyzed the measurement invariance for the MDE, GA, and PTSD factors by comparing sex, COVID-19 status, psychological care-seeking, and persistent bothersome symptoms for the samples that had experienced violence to examine the extent to which the items showed equivalent psychometric properties for comparisons. 

In the fifth step, we examined the difference between groups according to the latent means of dimensions (such as sex) for the total sample (MDE and GA) and the groups that had experienced violence (the six scales). In the final invariance models, we therefore constrained the latent variables of each group, comparing the fit of the models with and without constraints with the means. Significant chi-square values, CFI values of less than 0.010, and RMSEA value differences (Δ) of less than 0.015 indicated that the constrained means model was a model with restrictions with a good fit, meaning there were no significant differences between groups. 

In the sixth step, we undertook means, standard deviation, multivariate analysis, and the Cohen’s *d* effect analysis between the means of MDE and GA dimensions per the total, and between the means of the MDE, GA, PTSD, and their four PTSD factors in the samples that had experienced abuse between all the comparison groups (e.g., experience of abuse or persistent bothersome symptoms lasting). 

In the seventh step, we calculated the percentage of total subjects and those in the sample who had experienced violence who met the MDE, GA, and PTSD criteria as recommended by APA [6] and Goldberg et al. [10].

Finally, we integrated the overall model including the prediction between latent variables through a chi-square test and its fit indices through the SEM for the sample that had experienced abuse.

All analyses were conducted using lavaan 0.6–11 by the integrated development environment for R, RSTUDIO ^®^ 2022.07.1, from R Core Team [25] of the Foundation for Statistical Computing, Vienna, Austria. We also used SPSS ^®^ 25.0 software from IBM Corp. [26], In Armonk, NY, USA.

## 3. Results

### 3.1. Confirmatory Factorial Analyses, Cronbach’s Alpha, and Spearman Correlations

Results from the two-factor model for the total sample (*n* = 18,449) and each factor model for the sample that experienced abuse (*n* = 5712) are shown in Table 2. The data fit was adequate, with an *RMSEA* < 0.08, *SRMR* < 0.06, *TLI* and *CFI* > 0.90. 

As noted, the MDE model for the total sample (*n* = 18,449) yielded an $X^{2}$(32) = 2643.99, *p* < 0.001; an *RMSEA* = 0.067 (0.064–0.069), an *SRMR* = 0.023; a *CFI* = 0.975, and a *TLI* = 0.965. Its reliability (α) was 0.92. The GA model resulted in a $X^{2}$(5) = 350.57, *p* < 0.001; an *RMSEA* = 0.061 (0.056–0.067), an *SRMR* = 0.007, a *CFI* = 0.996, a *TLI* = 0.992, and an α = 0.94.

For the experience of abuse sample (*n* = 5712), the MDE model yielded an $X^{2}$(32) = 771.018, *p* < 0.001; an *RMSEA* = 0.064 (0.060–0.068), an *SRMR* = 0.026; a *CFI* = 0.972, a *TLI* = 0.960, and α = 0.90. The GA model yielded an $X^{2}$(5) = 87.133, *p* < 0.001, an *RMSEA* = 0.054 (0.044–0.064), an *SRMR* = 0.009, a *CFI* = 0.996, a *TLI* = 0.991, and α = 0.91. The four-factor PTSD model yielded an $X^{2}$(161) = 5648.340, *p* < 0.001, an *RMSEA* = 0.077 (0.076–0.079), an *SRMR* = 0.040, a *CFI* = 0.935, a *TLI* = 0.924, and an α = 0.96. 

For the total sample and the sample with experience of violence, the MI indicated the correlations required for the MDE dimension between items: *1. Sadness or depressed mood?* and *2. Discouraged because of how things are going in your life?* The MI also indicated the correlation between items *2* and *3. Loss of interest or pleasure*? and *2* with *4. Feeling worthless or not good enough*?

For the sample with experience of violence, the MI indicated the need to correlate items B1-Repeated, disturbing, and unwanted memories of the stressful experience with B4-feeling very upset when something reminded you of the stressful experience? (Reexperiencing dimension). MI also indicated the need to correlate item B4 with item C1 Do you avoid memories, thoughts, or feelings related to the stressful experience? (Avoidance scale).

Appendix C contains correlations between the scales, resulting in positive values from 0.519 to 0.857. The lowest correlation was obtained between reexperiencing and MDE for the sample that experienced abuse. Moreover, the highest correlation was obtained between NACM and hyperarousal scales for the same sample. These results suggest an overlap between NACM and hyperarousal, but we decided to regard them as independent dimensions because of previous empirical evidence [7]. Furthermore, correlation values and CFA model fit indices indicated that scale measurements are related yet have independent dimensions.

### 3.2. Measurement Invariance

Appendix D shows that changes in CFIs and RMSEA between the models (metric vs. configural) were acceptable, less than 0.010 and, 0.015, respectively, for all comparisons between the MDE and GA scales for the total sample. In other words, MDE and GA obtained an adequately fitted model in which loadings, thresholds, and unique variances were restricted to equality during the invariance measurement calculations indicating that no bias was found because of sex, COVID-19 status, psychological care-seeking, or the experience of violence for the total sample. The measurement invariance made an adequate means comparison between the groups referred to. The GA means between those seeking psychological care and those reporting the experience of violence proved different.

Appendix E, Appendix F, Appendix G and Appendix H show, by sex, COVID-19 status, psychological care-seeking, and persistent bothersome symptoms, respectively, that changes in CFIs and RMSEA between the models (i.e., metric vs. configural) were less than 0.010 and, 0.015, for all comparisons (MDE, GA and PTSD (total, and for re-experiencing, avoidance, NACM, and hyperarousal)) in the sample experiencing violence. 

Appendix E, Appendix F and Appendix G show that the restricted models with loadings, thresholds, and unique variance, restricted to equality, including the correlated items, obtained adequate fit indices in the invariance measurement calculation, indicating that no bias was found due to sex, COVID-19 status, or psychological care-seeking. This made it possible to compare MDE, GA, and PTSD (re-experiencing, avoidance, NACM, and hyperarousal) through the means of these groups for the sample that experienced violence. It should be noted that the average avoidance between sex groups, and reexperiencing, avoidance, NACM, hyperarousal, and GA for those seeking psychological care was different for this sample.

Appendix H shows that the restricted model with loadings, thresholds, and unique variance restricted to equality obtained adequate fit indices on the invariance measurement calculation for MDE and GA for persistent bothersome symptoms due to the experience of violence. The GA average for persistent bothersome symptoms was different for the sample with experience of abuse.

However, Appendix H also shows that we were obliged to freely estimate the unique variances of items B2, B5 (reexperiencing), C2 (avoidance), D1, D6, D7 (NACM), E2, E3, and E6 (hyperarousal), indicating their bias because of the persistence of bothersome symptoms. We freely estimated the loadings, thresholds, and unique variances of the items D2 (NACM) and E4 (hyperarousal), indicating the bias because of this group comparison. When considering the PTSD total, we freely estimated the unique variances of B2, B3, D1, D4, D6, D7, E1, E2, E3, and loadings, thresholds, and the unique variances of item E6 to compare the groups with persistent bothersome symptoms. After comparing the freely estimated parameters, we compared the means of interest. Note that the average of reexperiencing, avoidance, NACM, hyperarousal, total PTSD, and GA was different between those bothered by stress symptoms for over a month and those bothered by them for less than a month.

### 3.3. Comparison Groups Means

Table 3 shows the average (*M*) for all scales by sex, COVID-19 status, psychological care-seeking, and the experience of violence for the total sample. The same table also shows the means of the scales by sex, COVID-19 status, psychological care-seeking, and persistent bothersome symptoms for the sample with experience of abuse. The table also includes the *F* values, degrees of freedom, and *p* values, from the multivariate analyses, and the Cohen’s *d* effect size from comparisons. 

According to the restricted means models, Table 3 showed a low Cohen-d effect for the MDE between sex (*d* = −0.073), COVID-19 status (*d* = −0.157), psychological care-seeking (*d* = −0.198), and the experience of abuse (*d* = −0.190) for the total sample (*n* = 18,449). A low Cohen’s-*d* effect was also observed for the GA by sex (*d* = −0.173) and COVID-19 (*d* = −0.213) comparisons. Thus, GA resulted in moderate Cohen’s d effects by psychological care-seeking (*d* = −0.415) and the experience of violence (*d* = −0.356) in the group comparisons. 

For the sample with experience of violence (*n* = 5712), Table 3 shows that Cohen’s *d* effect sizes were close to zero when sex and COVID-19 groups for all scales (such as reexperiencing (*d* = −0.124)). Additionally, very low Cohen’s *d* effect sizes were observed in the comparison of psychological care-seeking and persistent bothersome symptoms groups for the MDE scale (*d* = −0.030 and *d* = −0.145, respectively).

Nevertheless, a low Cohen’s *d* effect was observed in reexperiencing (*d* = −0.279), avoidance (*d* = −0.273), NACM (*d* = −0.293), hyperarousal (*d* = −0.270), total PTSD (*d* = −0.297), and GA (*d* = −0.262) in the comparison of the psychological care-seeking groups and the sample that had experienced violence. In addition, a moderate Cohen’s *d* effect was obtained in reexperiencing (*d* = −0.543), avoidance (*d* = −0.472), NACM (*d* = −0.597), hyperarousal (*d* = −0.602), total PTSD (*d* = −0.592), and GA (*d* = −0.463) in the comparison of the group with persistent bothersome symptoms, with the same sub-sample.

### 3.4. Total Subjects’ MDE, GA, and MDE, GA, and PTDS Percentages in the Group That Had Experienced Violence

Table 4 shows the percentage of the total subjects and sample that had experienced abuse who met the MDE, GE, and PTSD screening criteria. The calculations indicated that 5179 (28.10%) of the total subjects met the MDE screening criterion. Of this group, 1265 (20.20%) men, 3914 (32.10%) women, 4150 (27.90%) subjects with non-COVID status, 1029 (28.90%) with COVID-19 status, 3728 (23.70%) non-care-seeking subjects, 1451 (53.60%) care-seeking subjects, 2415 (19.00%) who had not experienced violence, and 2764 (45.40%) who had experienced subjects met the MDE screening criteria.

A total of 7807 (42.30%) subjects met the GA screening criterion. Of these, 2091 (33.40%) men, 5716 (46.90%) women, 5776 (38.80%) with non-COVID status, 2031 (57.10%) with COVID-19 status, 5852 (37.20%) non-care-seeking subjects, 1955 (72.20%) care-seeking subjects, 4283 (33.60%) subjects who had not experienced violence, and 3524 (61.70%) who had experienced abuse met the GA screening criterion.

Regarding the percentages in the sample that had experienced violence, Table 4 indicates that 2764 (48.40%) subjects met the MDE screening criteria. Of this group, 591 (41.60%) men, 2173 (50.60%) women, 2239 (49.00%) with non-COVID status, 525 (45.90%) with COVID-19 status, 1837 (43.40%) non-care-seeking, 927 (62.60%) care-seeking, 263 (17.50%) subjects with less than a month of bothersome symptoms, and 2501 (59.40%) subjects with more than a month of bothersome symptoms met the MDE screening criteria.

Table 4 shows that 3524 (67.70%) subjects met the GA screening criterion. Of this group, 790 (55.70%) men, 2734 (63.70%) women, 2733 (59.80%) subjects with non-COVID status, 791 (69.10%) with COVID-19 status, 2385 (56.40%) non-care-seeking subjects, 1139 (76.90%) care-seeking subjects, 498 (33.10%) with less than a month of bothersome symptoms, and (71.90%) 3026 with more than a month of bothersome symptoms met the GA screening criterion.

Finally, Table 4 shows that 2858 (50%) of the sample that had experienced violence met the PTSD screening criterion. Of these, 627 (44.20%) men, 2231 (52.00%) women, 2259 (49.50%) subjects with non-COVID-19 status, 599 (52.40%) with COVID-19-status, 1880 (44.40%) non psychological care-seeking subjects, 978 (66.00%) psychological care-seeking subjects, and 2858 (67.90%) subjects with bothersome symptoms for over a month met the PTSD screening criterion.

### 3.5. Structural Equation Modeling

**Figure 1:**
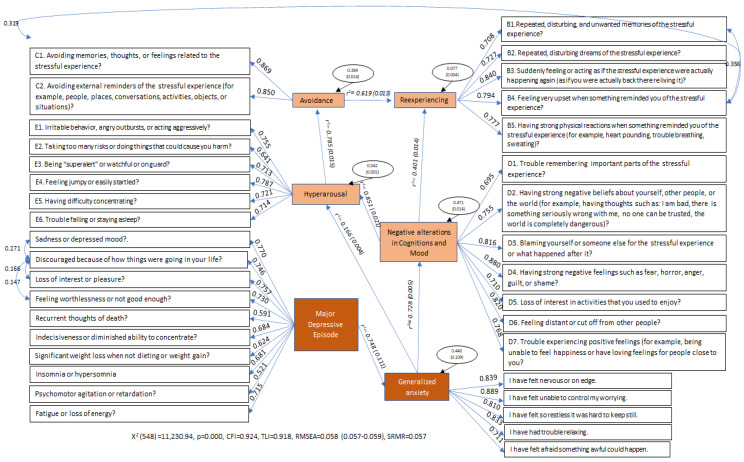
Latent variables from the SEM, their factor loadings, regression coefficients, residual variances, Chi-square, and fit indices for the sample with experience of abuse.

Figure 1 shows the resulting SEM for the sample with experience of abuse. The latent variables in the model included re-experiencing, avoidance, NACM, hyperarousal, MDE, and GA. Figure 1 shows items for each latent variable, their factorial loadings, the regression coefficients, and their residuals. The fit model resulted from 85 iterations with 82 parameters (*X*^2^(548) = 11,230.94, *p* = 0.000), with a *CFI* = 0.924, a *TLI* = 0.918, an *RMSEA* = 0.058 (0.057–0.059), and an *SRMR* = 0.057. The model therefore showed an adequate fit with factor loadings >0.40. Consequently, our results indicate that the latent reexperiencing variable was predicted by the avoidance one (*R*^2^ = 0.619) and NACM (*R*^2^ = 0.401); avoidance was predicted by the latent hyperarousal variable (*R*^2^ = 0.785); hyperarousal was predicted by NACM (*R*^2^ = 0.851) and GA (*R*^2^ = 0.166); and NACM was predicted by the latent GA variable (*R*^2^ = 0.728). Finally, GA was predicted by MDE (*R*^2^ = 0.748).

## 4. Discussion

The purpose of the study was to determine depressive, anxious, and posttraumatic stress symptoms related to the experience of violence during the COVID-19 pandemic in the general population by validating an electronic instrument, and obtaining its measurement invariance by sex, COVID-19 status, psychological care-seeking, the experience of violence, and persistent, bothersome stress conditions. An SEM was used to analyze the latent mental health variables path with the sample that had experienced abuse. 

Findings indicated that six latent variables—MDE, GA, and PTSD (reexperiencing, avoidance, NACM, and hyperarousal)—were validly and reliably measured to be considered in mental health screening in the general population in community and primary care settings [5,6,7,10]. 

The CFA yielded models with goodness of fit in six, separate dimensions, using the Chi-square, and *CFI*, *TLI*, *RMSEA*, and *SRMR* good indexes procedure [5,11,12]. CFA proved to be a systematic, reliable way to consider the validation of the latent variables, which were also validated through the SEM analysis. 

Depressive, anxious, and PTSD symptoms were related to latent variables, yet independent of each other, as noted by McDonald and Calhoun [27], Wilkins et al. [15], and Blevins et al. [7]. Goldberg et al. [10] posited these associations as the most common comorbidities in primary care settings. 

In keeping with our previous results, our findings indicate that the electronic depressive, anxiety, and posttraumatic stress tools resulted in measurement invariance, making it possible to compare groups by sex, COVID-19 status, psychological care-seeking, having experienced abuse, and bothersome stress symptoms [5]. However, care should be taken to consider the partial measurement invariance of PCL-5 while comparing bothersome stress symptoms lasting more or less than a month. 

Furthermore, it is important to note that associations between five items in the two latent variables (three MDE and two PTS symptoms) had been considered while their factor structure was validated. This limitation of the generalization of the model to other populations concurs with the DSM-5 [6] related to the diagnostic criterion. It means that the correlation between sadness or depressed mood and feeling discouraged due to how things are going in life, losing interest or pleasure, and feeling worthless or not good enough, while measuring depressive symptoms, points to the advisability of considering them together as the first step of the score to calculate an MDE diagnostic criterion. 

At the same time, the association between the stress items repeated disturbing, and unwanted memories of the stressful experience and feeling extremely upset when something reminded someone of the stressful experience, and avoiding memories, thoughts, or feelings related to the stressful experience also suggest the advisability of considering their over-2-option-response as part of the first step in the PTSD criterion. In other words, these associated items are part of the first 8-group of the PCL-5 which are considered essential to transitioning to the last 12 items of this tool, which coincides with the recommendation of the DSM-5 [6]. 

At the same time, the partial measurement invariance for PCL-5 for bothersome symptoms highlighted specific considerations for future studies. The findings pointed to the bias observed in the unique variances of items B5, C2, D1, D6, D7, E2, E3, and E6 while comparing groups by duration of stress symptoms. It is also suggested that future research should study bias because of the loadings, thresholds, and unique variances of items D2, and E4 in these comparisons. Out of the entire PCL-5 scale, the unique variances that should be considered in future studies are B2, B3, D1, D4, D6, D7, E1, E2, E3, and loadings, thresholds, and unique variance of item E6, when comparing the groups with persistent bothersome stress symptoms. It is worth noting that all the items referred to are contemplated in those 12 items, after the first eight items, when severe PTSD conditions are addressed in the DSM-5 [6]. Our findings appear to support Blevins et al.’s [7] proposal that PCL-5 is a reliable, valid tool, related to abuse experienced as a specific trauma, for assessing PTS symptoms. However, those biases should be considered, when comparing groups by persistent stress symptoms, because other factors may be affecting the measurement of these severe symptoms, such as the type of abuse involved, the pattern of the abuse, or the intensity of the abuse experience [3]. This action should guide decision-making regarding PTS symptom screening because of the characteristics of the population (community vs. specialized settings) when abuse occurs [15].

Finally, our findings suggested that GA symptoms of the general population were high in those reporting abuse experience, but also in those seeking psychological care. However, GA among men and women or with or without COVID-19 status was similar between subjects. Meanwhile, depressive symptoms were similar between women and men, those with and without COVID-19 status, those seeking psychological care or otherwise, and members of the general population who had or had not experienced violence. 

Moreover, it was observed that avoidance symptoms were high in women who had experienced violence, whether physical, emotional, or sexual. Moreover, PTS and GA symptoms were high in subjects who had experienced abuse and were seeking psychological care. Nevertheless, GA and stress symptoms between men and women, those with and without COVID-19 status, and subjects who had experienced violence or otherwise, were essentially the same. Meanwhile, men and women, with and without COVID-19 status, whether or not they were engaged in psychological care-seeking or had experienced violence reported similar levels of depressive symptoms. 

Our findings suggest high GA among subjects experiencing violence who reported having been bothered by their stress symptoms for over a month since their onset. However, depressive symptom levels were similar between subjects who had been bothered by their symptoms for less than a month and those who had had them for over a month since their onset. 

Furthermore, the averages of stress symptoms, lasting over a month since their onset, were high for those experiencing abuse. The experience of abuse related to the occurrence of PTS and GA made it possible to describe violence as a stressful event or trauma during the COVID-19 pandemic, as noted by Blevins et al. [7]. Even though future studies should verify clinical PTSD, our findings coincided with the stress symptoms reported by Blevins on 2015 among subjects who had experienced similar events. 

Our findings suggest that 28.10% and 42.30% of the general population have met the depressive and GA criteria. These percentages are slightly higher than the prevalence reported by Bourmistrova et al. [1] (20.84, and 11.11, respectively). Moreover, the proportions of women, those with COVID-19, who were engaged in psychological care-seeking, and had experienced violence, who had met the depressive and GA screening criteria, confirm the role of psychosocial factors as mental health determinants during the COVID-19 pandemic [1,5]. 

The high proportion of the general population who met depressive, GA, and stress screening criteria during the COVID-19 pandemic, points to the need to maintain early evaluation of these mental health conditions at the community and primary care level [28].

Moreover, a high percentage of the general population who met depressive and GA screening criteria were also seeking psychological care. The electronic tool is therefore an essential device in the early identification of symptoms and narrowing the treatment gap. It showed that 53.60% and 72.20% of the general population seeking psychological care, had met the criteria for depression and anxiety, respectively, and were therefore ready for treatment. 

In the same order of ideas, one in three of our general population reported having experienced violence, in keeping with what WHO [3] has identified as a worldwide social problem. The fact that the general population experiencing violence had met the depressive (45.40%) and GA (61.70%) screening criteria confirms the need to design strategies not only for treatment but also to prevent events that put people at risk of suffering violence. 

Our findings also indicate that 48.40%, 67.70%, and 50% of the population that had experienced violence met the criteria for MDE, GA, and PTSD, respectively. Again, these percentages were higher than the prevalence reported by Bourmistrova et al. [1] (20.84%, 11.11%, and 18.99%, respectively). In addition, Goldberg et al. [10] posited that high scores in depressive and anxious symptomatology are the most common risk factor in those seeking either specialized or regular care. 

A high percentage of the general population who had experienced violence, were seeking psychological care or had been bothered by their stress symptoms for over a month, met the three mental health screening criteria (62.60% and 59.40% for depressive symptoms, 76.90%, and 71.90% for GA; and 66.00%, and 67.90% for PTSD). These findings confirm that screening could be a way to reduce the treatment gap, and that people experiencing violence report severe mental health symptoms. 

Our findings link all PTS symptoms and GA to a specific event known as experience of abuse during the COVID-19 pandemic. Consequently, it is possible to identify mental health symptomatology related to a specific event early at the community level and in primary care settings, by monitoring these symptoms.

Finally, our findings suggest that there might be a series of symptoms we should consider when planning preventive actions, particularly when people have been experiencing violence. Depressive symptoms may predict anxiety, while anxiety predicts negative alterations in cognitions and mood, and hyperarousal symptoms. Hyperarousal may predict avoidance while negative mood alterations could lead to re-experiencing symptoms. Thus, as we suggested before, a sequence of mental health symptoms indicates a progressive path in which PTS symptoms were predicted by GA, and anxiety appeared to be the result of depressive symptoms [5]. 

The path of mental health symptoms might indicate that the general population suffering abuse is responding to a specific traumatic, stressful event through the general adaptation syndrome, proposed by De Camargo [29] and Selye [30]. In other words, the symptomatic transition described in this study seems to be characterized more by symptoms of exhaustive and resistance phases than symptoms of the awake phase. Before, we described symptoms of the awake stage caused by general events such as the COVID-19-pandemic lockdown, in which a state of alert could include freezing in response to a stressful event [4]. However, in the current study, the path of symptoms appears to be characterized by the stages of the resistance-exhaustion adaptation syndrome. De Camargo [29] describes the resistance phase as one in which high stress levels allow systems to be active for weeks or months, if not years. In the exhaustion phase, people report persistent tiredness, with reactions such as insomnia, fatigue, and lack of concentration, together with cardiovascular and metabolic reactions, endocrine responses, emotional problems, gastrointestinal issues, and vascular events.

Our findings suggest that such severe symptoms could be related to the violence experienced or persistent stress symptoms. Consequently, the hypothesis of De Camargo [29] and Selye [30] could be considered in future clinical studies exploring the consistency of the symptoms with the diagnosis criteria proposed on the DSM-5 [6].

In other words, since PTS and anxious symptoms were worse a month after their onset, we should consider the importance of the time that has elapsed between the occurrence of the abuse experienced as a traumatic event and the development of PTSD, in addition to anxiety symptoms [5]. The mental health path could also be considered in future studies to follow the clinical evolution of the PTSD criteria to be prevented, or to treat this disorder more effectively.

As we already suggested, current findings indicate that valid measures can be considered when physical, emotional, or sexual violence is reported, and people are aware that they require psychological care to help them cope with the etiology and development of depressive, anxious, and PTS symptoms at the early stages, and reduce the evidence-based treatment gap [16].

It is also essential to evaluate evidence-based psychological interventions as an effective means of reducing the treatment gap, while promoting mental health [31]. An electronic device, and valid, reliable mental health scales with measurement invariance, for comparing groups, is a gradual, successful procedure for transitioning to evidence-based treatment, and maximizing the scant mental health resources available in Mexico [32]. 

## 5. Conclusions

A device application achieved a validated electronic tool with measurement invariance by sex, COVID-19 status, psychological care-seeking, violence, and PTS-symptom groups. There was thus an association between mental health symptoms and the experience of violence in the general population during the COVID-19 pandemic. Experiencing abuse or already seeking psychological care were therefore conditions associated with depressive and anxiety symptoms. Experiencing violence was associated with generalized anxiety and severe posttraumatic stress symptoms when the discomfort had bothered them for over a month. Moreover, subjects who had experienced violence and had severe depressive, anxiety, and PTS symptoms seemed ready for treatment. Latent variables were independent of each other, and depression and anxiety predict PTS symptoms. Consequently, mental health problems in the community and primary healthcare settings during the COVID-19 pandemic pointed to the need for further studies required to provide consistent diagnoses of mental health disorders and evaluate the effect of remote psychological care to help reduce the treatment gap.

## 6. Limitations

Since the present study used measures of symptoms rather than diagnoses of mental health disorders, future studies should conduct a follow-up and assess their consistency with these diagnoses and evaluate the effect of remote psychological help. 

Since this study is not longitudinal, in the future, researchers could monitor the process and the time that has elapsed between the occurrence of traumatic events and the development of a PTSD, as well as other mental health risks, through measuring tools such as those used in this study. 

Another limitation was the need for a procedure that would help identify sources of bias from the items. Identifying the source of bias would make it possible to increase the accuracy of mental health symptom screening and halt the evolution of mental illness. 

Future studies should evaluate the comparability of the PCL-5, major depression symptoms, and Goldberg’s GA scale with its diagnostic utility and clinical PTSD diagnosis. They should also examine the sensitivity and specificity of these instruments. 

It would also be advisable to find a strategy to increase the representativeness of our sample. Since subjects participated voluntarily, we were unable to achieve this. Finally, subsequent studies should consider social determinants during the COVID-19 pandemic, such as age, unemployment, violence, and the use of drugs such as alcohol and tobacco to understand how they contribute to the early onset of mental health symptoms.

## Figures and Tables

**Table 1:** Distribution of subjects by sex, COVID-19 status, psychological care-seeking, and experience of abuse in the total sample, by sex, COVID-19, psychological care-seeking, and bothersome symptoms by experience of abuse. and average and standard deviation of subjects’ age by groups.

Men				Women				Total			
*n*		%		*n*		%		*n*		%	
6261		33.90		12,188		66.10		18,449		100%	
*Age Average (M)*		*Standard Deviation (SD)*		*Age Average (M)*		*Standard Deviation (SD)*		*Age Average (M)*		*Standard Deviation (SD)*	
33.60 *		11.15		33.15		10.93		33.30		11.00	
Non COVID-19 status		COVID-19 status		Non COVID-19 status		COVID-19 status		**Non COVID-19 status**		**COVID-19 status**	
*n*	%	*n*	%	*n*	%	*n*	%	*n*	%	*n*	%
5081	81.15	1180	18.85	9809	80.48	2379	19.52	14,890	80.71	3559	19.29
*Age (M)*	*SD*	*Age (M)*	*SD*	*Age (M)*	*SD*	*Age (M)*	*SD*	*Age (M)*	*SD*	*Age (M)*	*SD*
33.35 *	11.33	34.65	10.23	32.71 *	11.04	34.95	10.25	32.93	11.15	34.85	10.25
Non psychological care-seeking		Psychological care-seeking		Non psychological care-seeking		Psychological care-seeking		**Non psychological care-seeking**		**Psychological care-seeking**	
*n*	%	*n*	%	*n*	%	*n*	%	*n*	%	*n*	%
5494	87.75	767	12.25	10,249	84.09	1939	15.91	15,743	85.33	2706	14.67
*Age (M)*	*SD*	*Age (M)*	*SD*	*Age (M)*	*SD*	*Age (M)*	*SD*	*Age (M)*	*SD*	*Age (M)*	*SD*
33.82	11.18	31.99	10.78	33.47	10.89	31.43	10.99	33.59 *	10.99	31.59	10.93
Non violence experience		Violence experience		Non violence experience		Violence experience		**Non violence experience**		**Violence experience**	
*n*	%	*n*	%	*n*	%	*n*	%	*n*	%	*n*	%
4842	77.34	1419	22.66	7895	64.78	4293	35.22	12,737	69.04	5712	30.96
*Age (M)*	*SD*	*Age (M)*	*SD*	*Age (M)*	*SD*	*Age (M)*	*SD*	*Age (M)*	*SD*	*Age (M)*	*SD*
34.2	11.28	31.52	10.42	34.04	11.03	31.5	10.55	34.1 *	11.12	31.51	10.51
**Men**				**Women**				**Violence Experience Condition**			
*n*		%		*n*		%		*n*		%	
1419		24.80		4293		75.20		5712		100%	
*Age Average (M)*		*Standard Deviation (SD)*		*Age Average (M)*		*Standard Deviation (SD)*		*Age Average (M)*		*Standard Deviation (SD)*	
31.52		10.42		31.50		10.55		31.51		10.51	
Non COVID-19 status		COVID-19 status		Non COVID-19 status		COVID-19 status		**Non COVID-19 status**		**COVID-19 status**	
*n*	%	*n*	%	*n*	%	*n*	%	*n*	%	*n*	%
1123	79.10	296	20.90	3445	80.20	848	19.80	4568	80.00	1144	20.00
*Age (M)*	*SD*	*Age (M)*	*SD*	*Age (M)*	*SD*	*Age (M)*	*SD*	*Age (M)*	*SD*	*Age (M)*	*SD*
31.12	10.38	33.07	10.42	31.14	10.70	32.95	9.78	31.14 *	10.62	32.98	9.95
Non psychological care-seeking		Psychological care-seeking		Non psychological care-seeking		Psychological care-seeking		**Non psychological care-seeking**		**Psychological care-seeking**	
*n*	%	*n*	%	*n*	%	*n*	%	*n*	%	*n*	%
1076	75.80	343	24.20	3155	73.50	1138	26.50	4231	74.10	1481	25.90
*Age (M)*	*SD*	*Age (M)*	*SD*	*Age (M)*	*SD*	*Age (M)*	*SD*	*Age (M)*	*SD*	*Age (M)*	*SD*
31.73	10.41	30.87	10.42	31.79	10.54	30.69	10..53	31.78	10.51	30.73	10.50
Less than one month of bothering symptoms		More than one month of bothering symptoms		Less than one month of bothering symptoms		More than one month of bothering symptoms		**Less than one month of bothering symptoms**		**More than one month of bothering symptoms**	
*n*	%	*n*	%	*n*	%	*n*	%	*n*	%	*n*	%
443	31.20	976	68.80	1062	24.70	3231	75.30	1505	26.30	4207	73.70
*Age (M)*	*SD*	*Age (M)*	*SD*	*Age (M)*	*SD*	*Age (M)*	*SD*	*Age (M)*	*SD*	*Age (M)*	*SD*
33.11	10.94	30.8	10.09	32.68	11.02	31.11	10.36	32.81	11.00	31.04	10.30

Note. * significant differences < 0.05.

**Table 2:** Fit indices, Chi-square analysis, and Cronbach’s alpha, by scale, for the entire group of subjects (*n* = 18,449) and experience of violence (*n* = 5712).

	*n*=	*X*²	*df*	*p*≤	*RMSEA*	*Confident Interval*	*SRMR*	*CFI*	*TLI*	*Cronbach´Alpha*
MDE	18,449	2643.99	32	0.001	0.067	0.064–0.069	0.023	0.975	0.965	0.92
GA		350.57	5	0.001	0.061	0.056–0.067	0.007	0.996	0.992	0.94
MDE	5712	771.018	32	0.001	0.064	0.060–0.068	0.026	0.972	0.960	0.90
GA		87.133	5	0.001	0.054	0.044–0.064	0.009	0.996	0.991	0.91
PTSD		5648.340	161	0.001	0.077	0.076–0.079	0.040	0.935	0.924	0.96

Note. MDE = Major Depressive Episode, GA = Generalized Anxiety, PTSD = Postraumatic Stress Symptoms, *n* = number of subjects, *X*^2^ = Chi-Square, *df* = degree freedom, *RMSEA* = Root Mean Square Error of Approximation, *SRMR* = Standardized Root Mean Square Residual, *CFI* = Comparative Fit Index, *TLI* = Tucker-Lewis Index.

**Table 3:** Scale means sex, COVID-19 status, psychological care-seeking, and violence experience per total sample. Scale means sex, COVID-19, psychological care-seeking, and bothersome symptoms lasting per violence experience condition. This includes F, df, *p*-values, from the multi-variate analyses, and Cohen’s *d* effect size.

*n*=		TOTAL				ANOVA		*Cohen’s d Effect Size*
	Dimension	Men		Women				
		*M*	*SD*	*M*	*SD*	*F*(1, 18,447)	*p*-Value	
18,449	MDE	2.20	3.14	3.31	3.51	443.46	0.000	−0.073
	GA	39.30	34.59	50.90	34.26	470.83	0.000	−0.173
		**Non COVID-19 status**		**COVID-19 status**		**ANOVA**		***Cohen’s d effect size***
		*M*	*SD*	*M*	*SD*	*F*(1, 18,447)	*p*-value	
	MDE	2.87	3.43	3.21	3.41	28.41	0.000	−0.157
	GA	44.12	34.65	58.86	32.89	530.01	0.000	−0.213
		**Non Psychological care-seeking**		**Psychological care-seeking**		**ANOVA**		***Cohen’s d effect size***
		*M*	*SD*	*M*	*SD*	*F*(1, 18,447)	*p*-value	
	MDE	2.54	3.26	5.21	3.51	1507.38	0.000	−0.198
	GA	42.94	34.20	70.36	28.55	1552.48	0.000	−0.415
		**Non violence experience**		**Violence experience**		**ANOVA**		***Cohen’s d effect size***
		*M*	*SD*	*M*	*SD*	*F*(1, 18,447)	*p*-value	
	MDE	2.11	3.04	4.77	3.54	2723.96	0.000	−0.190
	GA	39.62	34.35	63.34	29.89	2031.71	0.000	−0.356
		**Total**						
		*M*			*SD*			
	MDE	*2.93*			*3.43*			
	GA	*46.96*			*34.81*			
***n*=**	**Dimension**	**Violence Experiences Sample´s Groups**				**ANOVA**		***Cohen’s d Effect Size***
		**Men**		**Women**				
		***M***	***SD***	***M***	***SD***	***F*(1, 5710)**	***p*-Value**	
5712	Rexperimentation	7.86	5.33	9.15	5.28	63.53	0.000	−0.124
	Avoidance	3.25	2.38	3.75	2.31	48.02	0.000	−0.114
	NACM	12.45	7.95	14.02	7.62	44.77	0.000	−0.111
	HyperArousal	10.51	6.58	11.90	6.20	51.93	0.000	−0.122
	PTSD	34.07	20.60	38.83	19.63	61.01	0.000	−0.123
	MDE	4.19	3.56	4.96	3.51	50.92	0.000	−0.013
	GA	59.30	30.97	64.67	29.41	34.54	0.000	−0.097
		**Non COVID-19 Status**		**COVID-19 Status**		**ANOVA**		***Cohen’s d effect size***
		*M*	*SD*	*M*	*SD*	*F*(1, 5710)	*p*-value	
	Rexperimentation	8.73	5.34	9.26	5.22	9.32	0.002	−0.053
	Avoidance	3.60	2.35	3.72	2.30	2.24	0.134	−0.031
	NACM	13.59	7.77	13.79	7.57	0.631	0.427	−0.012
	HyperArousal	11.42	6.33	12.12	6.26	11.34	0.001	−0.059
	PTSD	37.34	20.06	38.89	19.63	5.57	0.018	−0.036
	MDE	4.77	3.54	4.75	3.53	0.04	0.839	−0.071
	GA	61.99	30.16	68.70	28.16	46.44	0.000	−0.112
		**Non Psychological care-seeking**		**Psychological care-seeking**		**ANOVA**		***Cohen’s d effect size***
		*M*	*SD*	*M*	*SD*	*F*(1, 5710)	*p*-value	
	Rexperimentation	8.10	5.20	10.93	5.09	329.49	0.000	−0.279
	Avoidance	3.32	2.31	4.50	2.20	293.01	0.000	−0.273
	NACM	12.49	7.66	16.89	6.98	378.28	0.000	−0.293
	HyperArousal	10.74	6.33	13.89	5.70	284.72	0.000	−0.270
	PTSD	34.65	19.79	46.21	17.93	392.22	0.000	−0.297
	MDE	4.36	3.52	5.95	3.34	230.45	0.000	−0.030
	GA	59.68	30.37	73.78	25.79	254.7	0.000	−0.262
		**Less than one month bothering**		**More than one month bothering**		**ANOVA**		***Cohen’s d effect size***
		*M*	*SD*	*M*	*SD*	*F*(1, 5710)	*p*-value	
	Rexperimentation	4.98	4.67	10.21	4.84	1319.49	0.000	−0.543
	Avoidance	2.08	2.07	4.18	2.18	1048.65	0.000	−0.472
	NACM	6.92	7.18	16.03	6.39	2109.91	0.000	−0.597
	HyperArousal	6.04	5.99	13.53	5.17	2133.61	0.000	−0.602
	PTSD	20.03	18.67	43.95	16.32	2202.7	0.000	−0.592
	MDE	2.18	2.91	5.70	3.28	1349.02	0.000	−0.145
	GA	42.51	31.17	70.79	25.60	1199.85	0.000	−0.463
		**Total**						
		***M***			***SD***			
	Rexperimentation	8.83			5.32			
	Avoidance	3.62			2.34			
	NACM	13.63			7.73			
	HyperArousal	11.56			6.32			
	PTSD	37.65			19.98			
	MDE	4.77			3.54			
	GA	63.34			29.89			

Note. MDE = Major Depressive Episode, GA = Generalized Anxiety, NACM = Negative Alterations in Cognitions and Mood, PTSD = Posttraumatic Stress Disorder, *n* = number of subjects, M = Mean, SD = Standard Deviation, ANOVA = Analysis of Variance.

**Table 4:** Percentage of total subjects meeting MDE, GA criteria and, percentage meeting MDE, GA, and PTSD criteria by sex, COVID-19, psychological care-seeking, and persistent bothersome symptoms in the sample with experience of violence.

Total				Violence Experience Sample			
MDE							
*n*		%		*n*		%	
5179		28.10%		2764		48.40%	
**Men**		**Women**		**Men**		**Women**	
*n*	%	*n*	%	*n*	%	*n*	%
1265	20.20	3914	32.10	591	41.60	2173	50.60
**Non COVID-19 status**		**COVID-19 status**		**Non COVID-19 status**		**COVID-19 status**	
*n*	%	*n*	%	*n*	%	*n*	%
4150	27.90	1029	28.90	2239	49.00	525	45.90
**Non psychological care-seeking**		**Psychological care-seeking**		**Non psychological care-seeking**		**Psychological care-seeking**	
*n*	%	*n*	%	*n*	%	*n*	%
3728	23.70	1451	53.60	1837	43.40	927	62.60
**Non violence experience**		**Violence experience**		**Less than one month of bothering symptoms**		**More than one month of bothering symptoms**	
*n*	%	*n*	%	*n*	%	*n*	%
2415	19.00	2764	48.40	263	17.50	2501	59.40
**GA**							
*n*		%		*n*		%	
7807		42.30%		3524		61.70%	
**Men**		**Women**		**Men**		**Women**	
*n*	%	*n*	%	*n*	%	*n*	%
2091	33.40	*5716*	46.90	790	55.70	2734	63.70
**Non COVID-19 status**		**COVID-19 status**		**Non COVID-19 status**		**COVID-19 status**	
*n*	%	*n*	%	*n*	%	*n*	%
5776	38.80	2031	57.10	2733	59.80	791	69.10
**Non psychological care-seeking**		**Psychological care-seeking**		**Non psychological care-seeking**		**Psychological care-seeking**	
*n*	%	*n*	%	*n*	%	*n*	%
5852	37.2	1955	72.20	2385	56.40	1139	76.90
**Non violence experience**		**Violence experience**		**Less than one month of bothering symptoms**		**More than one month of bothering symptoms**	
*n*	%	*n*	%	*n*	%	*n*	%
4283	33.60	3524	61.70	498	33.10	3026	71.90
				**PTSD**			
				*n*		%	
				2858		50.00%	
				**Men**		**Women**	
				*n*	%	*n*	%
				627	44.20	2231	52.00
				**Non COVID-19 status**		**COVID-19 status**	
				*n*	%	*n*	%
				2259	49.50	599	52.40
				**Non psychological care-seeking**		**Psychological care-seeking**	
				*n*	%	*n*	%
				1880	44.40	978	66.00
				**Less than one month of bothering symptoms**		**More than one month of bothering symptoms**	
				*n*	%	*n*	%
				0	0.00	2858	67.90

Note. MDE = Major Depressive Episode, GA = Generalized Anxiety, PTSD = Posttraumatic Stress Disorder, n = number of subjects, M = Mean, % = Percentage of participants.

## Data Availability

The original contributions presented in the study are included in the article; further inquiries should be sent to the corresponding author/s.

## Appendix A. Survey Questionnaires

**Survey Questionnaires**												
**Socio-Demographic**		**Response Options**										
1	Sex	Men					Women					
2	COVID-19 status	No					Yes					
3	Remote Psychological Care-seeking	No					Yes					
Major Depression Episode		Response Options										
Subjects had experienced the symptoms in in the past twelve months.		Always	Nearly always		Sometimes			Rarely			Never	
1	Sadness or depressed mood?	1	2		3			4			5	
2	Discouraged because of how things were going in your life?											
3	Loss of interest or pleasure?											
4	Feeling worthlessness or not good enough?											
5	Recurrent thoughts of death?											
6	Indecisiveness or diminished ability to concentrate?											
7	Significant weight loss when not dieting or weight gain?											
8	Insomnia or hypersomnia											
9	Psychomotor agitation or retardation?											
10	Fatigue or loss of energy?											
11	Symptoms causing impairment in social, occupational, or other important areas of functioning?											
Generalized Anxiety		Response Options										
Subjects had felt anxious in the past two weeks.		Total absence of the symptom					Total presence of the symptom					
1	I have felt nervous or on edge.	0	1	2	3	4	5	6	7	8	9	10
2	I have felt unable to control my worrying.											
3	I have felt so restless it was hard to keep still.											
4	I have had trouble relaxing.											
5	I have felt afraid something awful could happen.											
	Posttraumatic Stress Disorder										Response Options	
Adapted Life Events Checklist											No	Yes
Experience of violence in past six months.												
1	Have you been a victim of physical violence (being attacked, hit, slapped, kicked, beaten up)										0	1
2	Was this physical abuse inflicted by a family member or your partner?											
3	Have you been a victim of emotional violence (insults, humiliation, screaming, dismissing, or similar experiences)											
4	Was this physical abuse inflicted by a family member or your partner?											
5	Have you been a victim of sexual violence (unwanted touching, sexual act through force, or threat of harm to maintain unwanted sexual activity)?											
6	Was this physical abuse inflicted by a family member or your partner?											
7	Have you been a victim of sexual harassment (e.g., soliciting intimate/physical contact in exchange for academic or work achievements, feeling continuous verbal-or-via-online threatened)?											
8	Have you been a victim of violence, assault with a weapon (for example, being shot, stabbed, threatened with a knife, gun, or bomb), kidnapped, or blackmailed?											
9	Has any relative been a victim of this kind of violence: assault with a weapon, threatened, kidnapped, severe assaulted, blackmailed, sexual assaulted, or murdered?											
10	Have you witnessed violence inflicted on another person (hitting, intimidating, humiliating, etc.)											
11	Was this violence in your family (e.g., from father to son, from any person to your partner, etc.?											
Please, choose the most severe event to answer the next questions.												
Bothersome symptoms in the past month.				Response Options								
Reexperiencing				Not al all		Slightly	Moderately		Quit a lot		Extremely	
B1.	Repeated, disturbing, and unwanted memories of the stressful experience?			0		1	2		3		4	
B2	Repeated, disturbing dreams of the stressful experience?											
B3	Suddenly feeling or acting as if the stressful experience were actually happening again (as if you were actually back there reliving it)?											
B4	Feeling very upset when something reminded you of the stressful experience?											
B5	Having strong physical reactions when something reminded you of the stressful experience (for example, heart pounding, trouble breathing, sweating)?											
Avoidance												
C1	Avoiding memories, thoughts, or feelings related to the stressful experience?											
C2	Avoiding external reminders of the stressful experience (for example, people, places, conversations, activities, objects, or situations)?											
Negative Alterations in Cognitions and Mood												
D1	Trouble remembering important parts of the stressful experience?											
D2	Having strong negative beliefs about yourself, other people, or the world (for example, having thoughts such as: I am bad, there is something seriously wrong with me, no one can be trusted, the world is completely dangerous)?											
D3	Blaming yourself or someone else for the stressful experience or what happened after it?											
D4	Having strong negative feelings such as fear, horror, anger, guilt, or shame?											
D5	Loss of interest in activities that you used to enjoy?											
D6	Feeling distant or cut off from other people?											
D7	Trouble experiencing positive feelings (for example, being unable to feel happiness or have loving feelings for people close to you?											
Hyperarousal												
E1	Irritable behavior, angry outbursts, or acting aggressively?											
E2	Taking too many risks or doing things that could cause you harm?											
E3	Being “superalert” or watchful or on guard?											
E4	Feeling jumpy or easily startled?											
E5	Having difficulty concentrating?											
E6	Trouble falling or staying asleep?											
Less/more-than-a-month-response				Response Options								
1	How long have the symptoms been bothering you?			Less than					More than a month			
				1					2			

## Appendix B

Latent variables from the structured equation model, their factor loadings, regression coefficients, residual variances, Chi-square, and fit indices for major depressive episode and generalized anxiety.

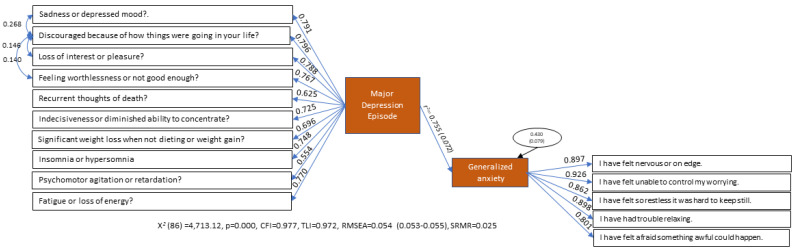

## Appendix C

Spearman Correlation coefficients between scales for the total (*n* = 18,449) and samples with experience of violence (*n* = 5712).

**Dimensions**	*n=*	MDE			GA			
MDE	18,449	1			0.701 **			
**Dimensions**	*n=*	Reexperiencing	Avoidance	NACM	Hyperarousal	MDE	GA	PTSD
Reexperiencing	5712	1	0.811 **	0.801 **	0.758 **	0.519 **	0.566 **	
Avoidance			1	0.713 **	0.636 **	0.456 **	0.475 **	
NACM				1	0.857 **	0.665 **	0.645 **	
Hyperarousal					1	0.631 **	0.687 **	
MDE						1	0.564 **	0.649 **
GA							1	0.673 **
PTSD								1
Note. ** *p* < 0.01; MDE = Major Depressive Episode, GA = Generalized Anxiety, NACM = Negative Alterations in Cognitions and Mood, PTSD = Posttraumatic Stress Disorder, n = number of participants.								

## Appendix D

Differences between chi-squares, df, measurement invariance fit indices (configural, metric, strong, and strict) in the models, and means, by sex, COVID-19, psychological care-seeking, and the experience of violence for depression (MDE) and generalized anxiety (GA) scales.

**Comparison**	**Models**	***X² (df)***	***CFI***	***TLI***	***RMSEA***	***SRMR***	**Δ*X² (Δdf)***	**Δ*X²’ s p*-Value**	**Δ*CFI***	**Δ*RMSEA***	**Δ*TLI***
**Sex**	**MDE**										
	Configural	3763.71 (64)	0.986	0.980	0.079	0.011					
	Metric	3991.62 (73)	0.985	0.982	0.076	0.021	227.91 (9)	0.000	0.001	0.003	0.002
	Strong	4397.65 (82)	0.984	0.982	0.076	0.022	406.02 (9)	0.000	0.001	0.000	0.000
	Strict	5212.04 (92)	0.980	0.981	0.078	0.023	814.39 (10)	0.000	0.004	0.002	0.001
	Strict.1 1–2, 2–3, 2–4	5255.03 (95)	0.980	0.981	0.077	0.023	857.39 (13)	0.000	0.004	0.001	0.001
	Means comparision	5338.83 (96)	0.980	0.981	0.077	0.033	83.80 (1)	0.000	0.000	0.000	0.000
	**GA**										
	Configural	359.37 (10)	0.996	0.992	0.062	0.008					
	Metric	372.94 (14)	0.996	0.994	0.053	0.010	13.58 (4)	0.009	0.000	0.009	0.002
	Strong	550.56 (18)	0.994	0.993	0.057	0.015	177.62 (4)	0.000	0.002	0.004	0.001
	Strict	775.16 (23)	0.991	0.992	0.060	0.017	224.60 (5)	0.000	−0.003	0.003	0.001
	Means comparison	1248.58 (24)	0.985	0.988	0.074	0.073	473.41 (1)	0.000	−0.006	0.014	0.004
**COVID-19**	**MDE**										
	Configural	3754.49 (64)	0.986	0.980	0.079	0.011					
	Metric	3915.00 (73)	0.985	0.982	0.076	0.018	160.51 (9)	0.000	0.001	0.003	0.002
	Strong	4078.40 (82)	0.985	0.983	0.073	0.019	163.40 (9)	0.000	0.000	0.003	0.001
	Strict	4525.84 (92)	0.983	0.983	0.072	0.020	447.44 (10)	0.000	0.002	0.001	0.000
	Strict.1 1–2, 2–3, 2–4	4546.27 (95)	0.983	0.984	0.071	0.020	467.87 (13)	0.000	0.002	0.002	0.001
	Means comparison	4821.78 (96)	0.982	0.983	0.073	0.042	275.51 (1)	0.000	0.001	0.002	0.001
	**GA**										
	Configural	374.18 (10)	0.996	0.991	0.063	0.008					
	Metric	419.49 (14)	0.995	0.993	0.056	0.012	45.31 (4)	0.000	0.001	0.007	0.002
	Strong	551.38 (18)	0.994	0.993	0.057	0.015	131.89 (4)	0.000	0.001	0.001	0.000
	Strict	586.38 (23)	0.993	0.994	0.052	0.017	35.00 (5)	0.000	0.001	0.005	0.001
	Means comparison	1096.12 (24)	0.987	0.989	0.07	0.067	509.74 (1)	0.000	0.006	0.018	0.005
**Seeking Psychological Care**	**MDE**										
	Configural	3709.38 (64)	0.986	0.980	0.079	0.012					
	Metric	3757.30 (73)	0.986	0.983	0.074	0.014	47.92 (9)	0.000	0.000	0.005	0.003
	Strong	4056.85 (82)	0.985	0.983	0.072	0.015	299.55 (9)	0.000	0.001	0.002	0.000
	Strict	5380.40 (92)	0.980	0.980	0.079	0.017	1323.54 (10)	0.000	0.005	0.007	0.003
	Strict.1 1–2, 2–3, 2–4	5437.96 (95)	0.979	0.981	0.078	0.017	1381.10 (13)	0.000	0.006	0.006	0.002
	Means comparison	5884.95 (96)	0.978	0.979	0.081	0.049	446.99 (1)	0.000	0.001	0.003	0.002
	**GA**										
	Configural	355.01 (10)	0.996	0.991	0.061	0.008					
	Metric	466.70 (14)	0.994	0.992	0.059	0.016	111.69 (4)	0.000	0.002	0.002	0.001
	Strong	536.58 (18)	0.994	0.993	0.056	0.019	69.88 (4)	0.000	0.000	0.003	0.001
	Strict	698.25 (23)	0.992	0.993	0.056	0.022	161.67 (5)	0.000	0.002	0.000	0.000
	Means comparison	2348.64 (24)	0.971	0.976	0.102	0.128	1650.40 (1)	0.000	0.021	0.046	0.017
**Violence Experience**	**MDE**										
	Configural	3818.73 (64)	0.986	0.980	0.080	0.012					
	Metric	3867.89 (73)	0.985	0.982	0.075	0.014	49.15 (9)	0.000	0.001	0.005	0.002
	Strong	4211.51 (82)	0.984	0.983	0.074	0.017	343.62 (9)	0.000	0.001	0.001	0.001
	Strict.1 9	6607.56 (91)	0.975	0.975	0.088	0.021	2396.05 (9)	0.000	0.009	0.014	0.008
	Strict.2 9 1–2, 2–3, 2–4	6696.86 (94)	0.975	0.976	0.087	0.021	2485.35 (12)	0.000	0.009	0.013	0.007
	Means comparison	7328.12 (95)	0.972	0.974	0.091	0.071	631.27 (1)	0.000	0.003	0.004	0.002
	**GA**										
	Configural	371.78 (10)	0.995	0.991	0.063	0.008					
	Metric	555.98 (14)	0.993	0.990	0.065	0.024	184.20 (4)	0.000	0.002	0.002	0.001
	Strong	568.95 (18)	0.993	0.992	0.058	0.024	12.97 (4)	0.011	0.000	0.007	0.002
	Strict	1160.86 (23)	0.986	0.988	0.073	0.031	591.91 (5)	0.000	0.007	0.015	0.004
	Means comparison	3240.17 (24)	0.96	0.967	0.121	0.17	2079.31 (1)	0.000	0.026	0.048	0.021
Note. MDE = Major Depressive Episode, GA = Generalized Anxiety, X^2^ = Chi-Square, df = degree freedom, CFI = Comparative Fit Index, TLI = Tucker-Lewis Index, RMSEA = Root Mean Square Error of Ap-proximation, SRMR = Standardized Root Mean Square Residual, ΔX^2^ (Δdf) = Chi Square subtraction of the model against the previous one (degree freedom subtraction of the model against the previous one), ΔX^2^’s *p*-value = *p*-value subtraction of the model against the previous one, ΔCFI = CFI subtraction of the model against the previous one, ΔRMSEA = RMSEA subtraction of the model against the previous one, ΔTLI = TLI subtraction of the model against the previous one.											

## Appendix E

Differences between chi-squares, df, measurement invariance fit indices (configural, metric, strong, and strict) in models, and means, by sex for all scales.

**Models**	***X² (df)***	***CFI***	***TLI***	***RMSEA***	***SRMR***	**Δ*X² (*Δ*df)***	**Δ*X²’s p*-Value**	**Δ*CFI***	**Δ*RMSEA***	**Δ*TLI***
**Reexperiencing**										
Configural	78.60 (8)	0.996	0.990	0.056	0.011					
Metric	81.69 (12)	0.996	0.993	0.045	0.012	3.08 (4)	0.544	0.000	0.011	0.003
Strong	104.58 (16)	0.995	0.994	0.044	0.016	22.89 (4)	0.000	0.001	0.001	0.001
Strict	116.99 (21)	0.995	0.995	0.040	0.018	12.41 (5)	0.030	0.000	0.004	0.001
Strict.1 1-4	121.14 (22)	0.994	0.995	0.040	0.017	16.56 (6)	0.011	0.001	0.004	0.001
Means comparision	179.65 (23)	0.991	0.992	0.049	0.044	58.51 (1)	0.000	0.003	0.009	0.003
**Avoidance**										
Strict	5.64 (2)	0.999	0.999	0.025	0.004					
Means comparison	57.18 (3)	0.989	0.992	0.080	0.047	51.55 (1)	0.000	0.010	0.055	0.007
**NACM**										
Configural	402.21 (22)	0.987	0.976	0.078	0.015					
Metric	406.75 (28)	0.988	0.981	0.069	0.017	4.54 (6)	0.604	0.001	0.009	0.005
Strong	427.70 (34)	0.987	0.984	0.064	0.018	20.96 (6)	0.002	0.001	0.005	0.003
Strict	453.74 (41)	0.986	0.986	0.059	0.019	26.03 (7)	0.000	0.001	0.005	0.002
Strict.1 1–3, 3–4, 2–6	455.92 (44)	0.986	0.987	0.057	0.018	28.21 (10)	0.002	0.001	0.007	0.003
Means comparison	502.89 (45)	0.985	0.986	0.060	0.036	46.97 (1)	0.000	0.001	0.003	0.001
**Hyperarousal**										
Configural	230.22 (16)	0.988	0.978	0.068	0.016					
Metric	239.17 (21)	0.988	0.983	0.060	0.019	8.95 (5)	0.111	0.000	0.008	0.005
Strong	318.49 (26)	0.984	0.982	0.063	0.022	79.32 (5)	0.000	0.004	0.003	0.001
Strict	341.40 (32)	0.983	0.984	0.058	0.023	22.91 (6)	0.001	0.001	0.005	0.002
Strict.1 4–5	341.41 (33)	0.983	0.985	0.057	0.023	22.91 (7)	0.002	0.001	0.006	0.003
Means comparison	395.78 (34)	0.98	0.983	0.061	0.04	54.37 (1)	0.000	0.003	0.004	0.002
**PTSD**										
Configural	6037.39 (320)	0.942	0.931	0.079	0.037					
Metric	6065.74 (339)	0.942	0.935	0.077	0.039	28.35 (19)	0.077	0.000	0.002	0.004
Strong	6182.06 (358)	0.941	0.937	0.075	0.040	116.32 (19)	0.000	0.001	0.002	0.002
Strict	6238.05 (378)	0.940	0.940	0.074	0.040	55.99 (20)	0.000	0.001	0.001	0.003
Strict.1	6254.38 (388)	0.940	0.941	0.073	0.040	72.32 (30)	0.000	0.001	0.002	0.004
Means comparison	6314.36 (389)	0.94	0.941	0.073	0.048	59.98 (1)	0.000	0.000	0.000	0.000
**MDE**										
Configural	1097.88 (64)	0.980	0.972	0.075	0.020					
Metric	1137.33 (73)	0.980	0.975	0.071	0.025	39.45 (9)	0.000	0.000	0.004	0.003
Strong	1245.42 (82)	0.978	0.976	0.070	0.027	108.09 (9)	0.000	0.002	0.001	0.001
Strict	1276.94 (92)	0.977	0.978	0.067	0.028	31.52 (10)	0.000	0.001	0.003	0.002
Strict.1 1–2, 2–3, 2–4	1294.53 (95)	0.977	0.978	0.066	0.028	49.11 (13)	0.000	0.001	0.004	0.002
Means comparison	1295.19 (96)	0.977	0.978	0.066	0.028	0.65 (1)	0.419	0.000	0.000	0.000
**GA**										
Configural	98.88 (10)	0.995	0.991	0.056	0.01					
Metric	104.80 (14)	0.995	0.993	0.048	0.013	5.93 (4)	0.205	0.000	0.008	0.002
Strong	143.09 (18)	0.993	0.993	0.049	0.016	38.29 (4)	0.000	0.002	0.001	0.000
Strict	160.82 (23)	0.993	0.994	0.046	0.017	17.73 (5)	0.003	0.000	0.003	0.001
Means comparison	197.00 (24)	0.991	0.992	0.05	0.033	36.18 (1)	0.000	0.002	0.004	0.002
Note. NACM = Negative Alterations in Cognitions and Mood, PTSD = Posttraumatic Stress Disorder, MDE = Major Depressive Episode, GA = Generalized Anxiety, X^2^ = Chi-Square, df = degree freedom, CFI = Comparative Fit Index, TLI = Tucker-Lewis Index, RMSEA = Root Mean Square Error of Ap-proximation, SRMR = Standardized Root Mean Square Residual, ΔX^2^ (Δdf) = Chi Square subtraction of the model against the previous one (degree freedom subtraction of the model against the previous one), ΔX^2^´s *p*-value = *p*-value subtraction of the model against the previous one, ΔCFI = CFI subtraction of the model against the previous one, ΔRMSEA = RMSEA subtraction of the model against the previous one, ΔTLI = TLI subtraction of the model against the previous one.										

## Appendix F

Differences between chi-squares, df, measurement invariance fit indices (configural, metric, strong, and strict) in models, and means, by COVID-19 status, for all scales.

**Models**	***X² (df)***	***CFI***	***TLI***	***RMSEA***	***SRMR***	**Δ*X² (Δdf)***	**Δ*X²’s p*-Value**	**Δ*CFI***	**Δ*RMSEA***	**Δ*TLI***
**Reexperiencing**										
Configural	80.49 (8)	0.996	0.990	0.056	0.011					
Metric	87.44 (12)	0.996	0.993	0.047	0.014	6.95 (4)	0.139	0.000	0.009	0.003
Strong	104.88 (16)	0.995	0.994	0.044	0.016	17.44 (4)	0.002	0.001	0.003	0.001
Strict	107.62 (21)	0.995	0.995	0.038	0.016	2.73 (5)	0.741	0.000	0.006	0.001
Strict.1 1–4	114.48 (22)	0.995	0.995	0.038	0.016	9.6 (6)	0.143	0.000	0.006	0.001
Means comparision	124.07 (23)	0.994	0.995	0.039	0.021	9.59 (1)	0.002	0.001	0.001	0.000
**Avoidance**										
Strict	3.67 (2)	1.000	1.000	0.017	0.004					
Means comparison	7.09 (3)	0.999	0.999	0.022	0.011	3.42 (1)	0.064	0.001	0.005	0.001
**NACM**										
Configural	389.96 (22)	0.988	0.977	0.077	0.015					
Metric	398.23 (28)	0.988	0.982	0.068	0.018	8.27 (6)	0.219	0.000	0.009	0.005
Strong	411.62 (34)	0.988	0.985	0.062	0.019	13.40 (6)	0.037	0.000	0.006	0.003
Strict	434.34 (41)	0.987	0.987	0.058	0.019	22.72 (7)	0.002	0.001	0.004	0.002
Strict.1 1–3, 3–4, 2–6	441.22 (44)	0.987	0.988	0.056	0.019	29.59 (10)	0.001	0.001	0.006	0.003
Means comparison	441.73 (45)	0.987	0.988	0.056	0.019	0.52 (1)	0.473	0.000	0.000	0.000
**Hyperarousal**										
Configural	233.22 (16)	0.988	0.978	0.069	0.016					
Metric	242.83 (21)	0.988	0.983	0.061	0.018	9.61 (5)	0.087	0.000	0.008	0.005
Strong	284.41 (26)	0.986	0.984	0.059	0.020	41.58 (5)	0.000	0.002	0.002	0.001
Strict	290.04 (32)	0.986	0.987	0.053	0.020	5.63 (6)	0.466	0.000	0.006	0.003
Strict.1 4–5	290.42 (33)	0.986	0.987	0.052	0.020	6.01 (7)	0.539	0.000	0.007	0.003
Means comparison	301.84 (34)	0.985	0.987	0.053	0.025	11.42 (1)	0.001	0.001	0.001	0.000
**PTSD**										
Configural	5959.59 (320)	0.943	0.932	0.079	0.037					
Metric	5985.48 (339)	0.943	0.936	0.076	0.038	25.89 (19)	0.133	0.000	0.003	0.004
Strong	6095.98 (358)	0.942	0.938	0.075	0.038	110.50 (19)	0.000	0.001	0.001	0.002
Strict	6131.40 (378)	0.942	0.941	0.073	0.038	35.41 (20)	0.018	0.000	0.002	0.003
Strict.1	6149.56 (388)	0.942	0.943	0.072	0.038	53.58 (30)	0.005	0.000	0.003	0.005
Means comparison	6154.34 (389)	0.942	0.943	0.072	0.039	4.77 (1)	0.029	0.000	0.000	0.000
**MDE**										
Configural	1110.58 (64)	0.980	0.972	0.076	0.020					
Metric	1133.28 (73)	0.980	0.975	0.071	0.023	22.70 (9)	0.007	0.000	0.005	0.003
Strong	1189.32 (82)	0.979	0.977	0.069	0.024	56.04 (9)	0.000	0.001	0.002	0.002
Strict	1205.40 (92)	0.979	0.979	0.065	0.025	16.07 (10)	0.098	0.000	0.004	0.002
Strict.1 1–2, 2–3, 2–4	1209.07 (95)	0.979	0.980	0.064	0.025	19.74 (13)	0.102	0.000	0.005	0.003
Means comparison	1226.84 (96)	0.978	0.98	0.064	0.029	17.78 (1)	0.000	0.001	0.000	0.000
**GA**										
Configural	101.90 (10)	0.995	0.99	0.057	0.009					
Metric	108.09 (14)	0.995	0.993	0.048	0.012	6.20 (4)	0.185	0.000	0.009	0.003
Strong	147.36 (18)	0.993	0.992	0.050	0.016	39.27 (4)	0.000	0.002	0.002	0.001
Strict	165.39 (23)	0.992	0.993	0.047	0.017	18.03 (5)	0.003	0.001	0.003	0.001
Means comparison	209.47 (24)	0.99	0.992	0.052	0.037	44.07 (1)	0.000	0.002	0.005	0.001
Note. NACM = Negative Alterations in Cognitions and Mood, PTSD = Posttraumatic Stress Disorder, MDE = Major Depressive Episode, GA = Generalized Anxiety, X² = Chi-Square, df = degree freedom, CFI = Comparative Fit Index, TLI = Tucker-Lewis Index, RMSEA = Root Mean Square Error of Ap-proximation, SRMR = Standardized Root Mean Square Residual, ΔX²(Δdf) = Chi Square subtraction of the model against the previous one (degree freedom subtraction of the model against the previous one), ΔX²’s *p*-value= *p*-value subtraction of the model against the previous one, ΔCFI = CFI subtraction of the model against the previous one, ΔRMSEA = RMSEA subtraction of the model against the previous one, ΔTLI = TLI subtraction of the model against the previous one.										

## Appendix G

Differences between chi-squares, df, measurement invariance fit indices (configural, metric, strong, and strict) in models, and means by psychological care-seeking, for all scales.

**Models**	***X² (df)***	***CFI***	***TLI***	***RMSEA***	***SRMR***	***ΔX² (Δdf)***	***Δ X²’s p*-value**	***ΔCFI***	***ΔRMSEA***	***ΔTLI***
**Reexperiencing**										
Configural	85.93 (8)	0.995	0.989	0.058	0.011					
Metric	90.72 (12)	0.995	0.992	0.048	0.015	4.08 (4)	0.308	0.000	0.010	0.003
Strong	109.12 (16)	0.995	0.993	0.045	0.018	18.40 (4)	0.001	0.000	0.003	0.001
Strict	135.44 (21)	0.993	0.994	0.044	0.021	26.31 (5)	0.000	0.002	0.001	0.001
Strict.1 1–4	136.97 (22)	0.993	0.994	0.043	0.021	27.84 (6)	0.000	0.002	0.002	0.001
Means comparision	464.42 (23)	0.974	0.978	0.082	0.102	327.45 (1)	0.000	0.019	0.039	0.016
**Avoidance**										
Strict	7.40 (2)	0.999	0.999	0.031	0.010					
Means comparison	293.81 (3)	0.937	0.958	0.184	0.125	286.41 (1)	0.000	0.062	0.153	0.041
**NACM**										
Configural	389.85 (22)	0.987	0.976	0.077	0.016					
Metric	415.01 (28)	0.987	0.980	0.070	0.022	25.17 (6)	0.000	0.000	0.007	0.004
Strong	444.82 (34)	0.986	0.983	0.065	0.023	29.80 (6)	0.000	0.001	0.005	0.003
Strict	533.19 (41)	0.983	0.983	0.065	0.027	88.37 (7)	0.000	0.003	0.000	0.000
Strict.1 1–3, 3–4, 2–6	548.74 (44)	0.983	0.983	0.063	0.027	103.92 (10)	0.000	0.003	0.002	0.000
Means comparison	939.36 (45)	0.969	0.971	0.083	0.104	390.62 (1)	0.000	0.014	0.020	0.012
**Hyperarousal**										
Configural	239.24 (16)	0.987	0.976	0.070	0.016					
Metric	257.21 (21)	0.987	0.981	0.063	0.022	17.79 (5)	0.003	0.000	0.007	0.005
Strong	287.75 (26)	0.985	0.983	0.059	0.024	30.53 (5)	0.000	0.002	0.004	0.002
Strict	361.08 (32)	0.981	0.982	0.060	0.028	73.34 (6)	0.000	0.004	0.001	0.001
Strict.1 4–5	361.26 (33)	0.981	0.983	0.059	0.028	73.52 (7)	0.000	0.004	0.000	0.000
Means comparison	676.27 (34)	0.964	0.968	0.081	0.091	315.00 (1)	0.000	0.017	0.022	0.015
**PTSD**										
Configural	5938.49 (320)	0.941	0.930	0.078	0.038					
Metric	6012.07 (339)	0.940	0.933	0.077	0.043	73.58 (19)	0.000	0.001	0.001	0.003
Strong	6085.46 (358)	0.940	0.936	0.075	0.043	73.39 (19)	0.000	0.000	0.002	0.003
Strict	6329.21 (378)	0.937	0.937	0.074	0.044	243.74 (20)	0.000	0.003	0.001	0.001
Strict.1	6367.49 (388)	0.937	0.938	0.073	0.044	282.03 (30)	0.000	0.003	0.002	0.002
Means comparison	6783.51 (389)	0.932	0.934	0.076	0.083	416.02 (1)	0.000	0.005	0.003	0.004
**MDE**										
Configural	1106.35 (64)	0.980	0.972	0.076	0.020					
Metric	1146.92 (73)	0.979	0.974	0.072	0.025	40.56 (9)	0.000	0.001	0.004	0.002
Strong	1230.37 (82)	0.978	0.976	0.070	0.026	83.46 (9)	0.000	0.001	0.002	0.002
Strict	1252.41 (92)	0.978	0.978	0.066	0.027	22.04 (10)	0.015	0.000	0.004	0.002
Strict.1 1–2, 2–3, 2–4	1255.51 (95)	0.978	0.979	0.065	0.027	25.14 (13)	0.022	0.000	0.005	0.003
Means comparison	1259.82 (96)	0.978	0.979	0.065	0.028	4.31 (1)	0.038	0.000	0.000	0.000
**GA**										
Configural	92.88 (10)	0.995	0.991	0.054	0.01					
Metric	109.64 (14)	0.995	0.992	0.049	0.017	16.76 (4)	0.002	0.000	0.005	0.001
Strong	140.34 (18)	0.993	0.992	0.049	0.020	30.70 (4)	0.000	0.002	0.000	0.000
Strict	169.61 (23)	0.992	0.993	0.047	0.022	29.26 (5)	0.000	0.001	0.002	0.001
Means comparison	473.48 (24)	0.975	0.979	0.081	0.097	303.88 (1)	0.000	0.017	0.034	0.014
Note. NACM = Negative Alterations in Cognitions and Mood, PTSD = Posttraumatic Stress Disorder, MDE = Major Depressive Episode, GA = Generalized Anxiety, X² = Chi-Square, df = degree freedom, CFI = Comparative Fit Index, TLI = Tucker-Lewis Index, RMSEA = Root Mean Square Error of Ap-proximation, SRMR = Standardized Root Mean Square Residual, ΔX²(Δdf) = Chi Square subtraction of the model against the previous one (degree freedom subtraction of the model against the previous one), ΔX²’s *p*-value= *p*-value subtraction of the model against the previous one, ΔCFI = CFI subtraction of the model against the previous one, ΔRMSEA = RMSEA subtraction of the model against the previous one, ΔTLI = TLI subtraction of the model against the previous one.										

## Appendix H

Differences between models´ chi-squares, df, measurement invariance fit indices (configural, metric, strong, and strict), and means per bothering-symptoms lasting, for all scales.

**Models**	***X² (df)***	***CFI***	***TLI***	***RMSEA***	***SRMR***	**Δ*X² (Δdf)***	**Δ*X²’s p*-Value**	**Δ*CFI***	**Δ*RMSEA***	**Δ*TLI***
**Reexperiencing**										
Configural	82.84 (8)	0.995	0.987	0.057	0.013					
Metric	103.00 (12)	0.994	0.990	0.052	0.022	20.17 (4)	0.000	0.001	0.005	0.003
Strong	105.86 (16)	0.994	0.992	0.044	0.024	2.85 (4)	0.583	0.000	0.008	0.002
Strict.2 B2, B5	200.83 (19)	0.988	0.987	0.058	0.031	94.97 (3)	0.000	0.006	0.014	0.005
Strict.2 B2, B5 1–4	203.00 (20)	0.988	0.988	0.057	0.032	97.14 (4)	0.000	0.006	0.013	0.004
Means comparision	1343.17 (21)	0.911	0.915	0.148	0.260	1140.17 (1)	0.000	0.077	0.091	0.073
**Avoidance**										
Strict.1 C2	8.30 (1)	0.998	0.997	0.051	0.014					
Means comparison	919.49 (2)	0.780	0.780	0.401	0.259	911.19 (1)	0.000	0.218	0.350	0.217
**NACM**										
Configural	381.75 (22)	0.985	0.971	0.076	0.020					
Metric.1 D2	530.59 (27)	0.978	0.966	0.081	0.047	148.84 (5)	0.000	0.007	0.005	0.005
Strong.1 D2	541.03 (32)	0.978	0.971	0.075	0.048	10.44 (5)	0.064	0.000	0.006	0.005
Strict.4 D1 D2 D6 D7	695.33 (35)	0.972	0.966	0.081	0.051	154.30 (3)	0.000	0.006	0.006	0.005
Strict.4 D1 D2 D6 D7 1–3, 3–4, 2–6	776.16 (38)	0.968	0.965	0.082	0.052	235.13 (6)	0.000	0.010	0.007	0.006
Means comparison	2079.98 (39)	0.912	0.906	0.135	0.319	1303.83 (1)	0.000	0.056	0.053	0.059
**Hyperarousal**										
Configural	264.38 (16)	0.982	0.966	0.074	0.023					
Metric.1 E4	400.38 (20)	0.972	0.958	0.082	0.046	136.01 (4)	0.000	0.010	0.008	0.008
Strong.1 E4	455.68 (24)	0.968	0.960	0.079	0.052	55.30 (4)	0.000	0.004	0.003	0.002
Strict.4 E2 E3 E4 E6	529.14 (26)	0.963	0.957	0.082	0.055	73.46 (2)	0.000	0.005	0.003	0.003
Strict.4 E2 E3 E4 E6 4–5	529.14 (27)	0.963	0.959	0.081	0.055	73.46 (3)	0.000	0.005	0.002	0.001
Means comparison	1800.44 (28)	0.869	0.86	0.149	0.303	1271.3 (1)	0.000	0.094	0.068	0.099
**PTSD**										
Configural	5732.45 (320)	0.931	0.918	0.077	0.045					
Metric.1 E6	6485.84 (338)	0.922	0.912	0.080	0.081	753.39 (18)	0.000	0.009	0.003	0.006
Strong.1 E6	6657.60 (356)	0.920	0.914	0.079	0.084	171.75 (18)	0.000	0.002	0.001	0.002
Strict.10 E1, 2, 3, 6 B2, 3, D1,4,6,7	7284.23 (366)	0.912	0.909	0.081	0.092	626.63 (10)	0.000	0.008	0.002	0.005
Strict.10.1 E1, 2, 3, 6 B2, 3, D1,4,6,7	7456.46 (376)	0.910	0.909	0.081	0.091	798.86 (20)	0.000	0.010	0.002	0.005
Means comparison	8772.77 (377)	0.893	0.892	0.088	0.337	1316.31 (1)	0.000	0.017	0.007	0.017
**MDE**										
Configural	1170.85 (64)	0.978	0.969	0.078	0.022					
Metric	1203.49 (73)	0.977	0.972	0.074	0.026	32.65 (9)	0.000	0.001	0.004	0.003
Strong	1359.00 (82)	0.974	0.973	0.074	0.029	155.51 (9)	0.000	0.003	0.000	0.001
Strict	1868.53 (92)	0.964	0.965	0.082	0.032	509.53(10)	0.000	0.010	0.008	0.008
Strict.1 1–2, 2–3, 2–4	1909.30 (95)	0.964	0.966	0.082	0.031	550.29 (13)	0.000	0.010	0.008	0.007
Means comparison	1983.01 (96)	0.962	0.965	0.083	0.049	73.72 (1)	0.000	0.002	0.001	0.001
**GA**										
Configural	110.99 (10)	0.994	0.987	0.059	0.011					
Metric	163.04 (14)	0.991	0.987	0.061	0.028	52.05 (4)	0.000	0.003	0.002	0.000
Strong	175.07 (18)	0.990	0.989	0.055	0.030	12.03 (4)	0.017	0.001	0.006	0.002
Strict	249.23 (23)	0.986	0.988	0.059	0.039	74.16 (5)	0.000	0.004	0.004	0.001
Means comparison	1069.56 (24)	0.934	0.945	0.124	0.196	820.33 (1)	0.000	0.052	0.065	0.043
Note. NACM = Negative Alterations in Cognitions and Mood, PTSD = Posttraumatic Stress Disorder, MDE = Major Depressive Episode, GA = Generalized Anxiety, X² = Chi-Square, df = degree freedom, CFI = Comparative Fit Index, TLI = Tucker-Lewis Index, RMSEA = Root Mean Square Error of Ap-proximation, SRMR = Standardized Root Mean Square Residual, ΔX²(Δdf) = Chi Square subtraction of the model against the previous one (degree freedom subtraction of the model against the previous one), Δ X²’s *p*-value= *p*-value subtraction of the model against the previous one, ΔCFI = CFI subtraction of the model against the previous one, ΔRMSEA = RMSEA subtraction of the model against the previous one, ΔTLI = TLI subtraction of the model against the previous one.										
